# Discovery and Validation of Grain Shape Loci in U.S. Rice Germplasm Through Haplotype Characterization

**DOI:** 10.3389/fgene.2022.923078

**Published:** 2022-09-12

**Authors:** Brijesh Angira, Tommaso Cerioli, Adam N. Famoso

**Affiliations:** ^1^ H. Rouse Caffey Rice Research Station, Louisiana State University Agricultural Center, Baton Rouge, LA, United States; ^2^ School of Plant, Environmental and Soil Science, Louisiana State University, Baton Rouge, LA, United States

**Keywords:** rice, grain shape, grain length, QTL, haplotypes, high-throughput KASP marker, SNP

## Abstract

Rice grain shape is a major determinant of rice market value and the end-use. We mapped quantitative trait loci (QTL) for grain shape traits in a bi-parental recombinant inbred line population (Trenasse/Jupiter) and discovered two major grain length QTLs—*qGL3.1* and *qGL7.1*. Previously, a major grain shape gene *GS3* was reported in the *qGL3.1* region and grain length gene *GL7* was reported to be encompassing *qGL7.1* locus. The re-sequencing SNP data on the International Rice Research Institute (IRRI) 3K Rice Genome Project (RGP) panel were obtained from the IRRI SNP-Seek database for both genes and haplotype diversity was characterized for each gene in this diverse panel. United States rice germplasm was not well represented in the IRRI 3K RGP database. Therefore, a minimum SNP set was identified for each gene that could differentiate all the characterized haplotypes. These haplotypes in the 3K RGP panel were screened across 323 elite U.S. genotypes using the minimum SNP set. The screening of haplotypes and phenotype association confirmed the role of *GS3* under *qGL3.1*. However, screening of the *GL7* haplotypes in the U.S. germplasm panel showed that *GL7* did not play a role in *qGL7.1*, and in addition, *GL7.1* did not segregate in the Trenasse/Jupiter RIL population. This concluded that *qGL7.1* is a novel QTL discovered on chr7 for grain shape in the Trenasse/Jupiter RIL population. A high-throughput KASP-based SNP marker for each locus (*GS3* and *qGL7.1*) was identified and validated in elite U.S. rice germplasm to be used in an applied rice breeding program.

## Introduction

Rice is a major source of calories for human diets, and the direct consumption of the grain makes grain quality a major component for rice industries. Grain shape is a major grain quality component of rice and directly influences market value. Consumers make decisions based on the cooking quality and presumed taste of rice depending on its grain length, grain width, aroma, and whiteness. Regions, their cultures, and rice cuisines also drive the preference for different types of rice. Rice is classified by grain types based on the length and width of the grains. However, there is no global standard for rice classification based on shape and size. In the United States, medium grain is predominantly grown in California, accounting for 70% of the U.S. medium-grain rice acres. Long grain is predominantly grown in the southern United States, accounting for 99% of the U.S. long-grain rice acres (USDA and National Agricultural Statistics Service, 2021).

Two primary subspecies or varietal groups—*Indica* and *Japonica*—represent two genetically distinct genetic pools of *O. sativa* (Oka, 1988; Wang and Tanksley, 1989; Sun et al., 2002; Childs, 2014). The varietal group *Indica* is adapted and predominantly grown in tropical latitudes; whereas, *Japonica* is grown in both temperate and tropical climates (Mackill and Lei, 1997). Each varietal group can be further divided into genetic subpopulations. The varietal group *Japonica* comprises *temperate japonica*, *tropical japonica*, and *aromatic* subpopulations, while *Indica* comprises *indica* and *aus* subpopulations (Second, 1985; Glaszmann, 1987; Garris et al., 2005; Caicedo et al., 2007; McCouch et al., 2016). Significant variation for grain length and shape exists between and within each group, and certain subpopulations are associated with specific grain classes. For example, the *temperate japonica* subpopulation is associated with short and round grains, *aus* with short and slender grains, *indica* with long, slender grains, and *aromatic* with both very short and extra-long, fragrant rice (Juliano and Villareal, 1993). Almost all cultivated varieties in the United States belong to the *Japonica* varietal group. United States long-grain rice is *tropical japonica*, while U.S. medium-grain rice is a mix of *temperate* and *tropical japonica* (Lu et al., 2005).

Grain shape traits are controlled by variation in naturally occurring genes that are often first identified as quantitative trait loci (QTLs). Some grain shape genes in rice are also responsible for grain weight and yield. Chen et al. (2022) reported *KRN2/OsKRN2* genes in rice and maize and their study showed that a complete loss of function allele of these genes increased grain numbers and yield without a negative impact on other agronomic traits. Many major genes and QTLs have been reported for grain shape traits in rice (Sun et al., 2018). The first grain size QTL to be cloned and characterized was *GS3* (Fan et al., 2006; Takano-Kai et al., 2009). *GS3* encodes a heterotrimeric G protein that interacts with other G-protein subunits as part of a signaling pathway that regulates grain size in rice (Botella, 2012; Sun et al., 2018). The wild-type *GS3* protein produces medium grain, a loss-of-function allele results in long grain, while truncated forms lacking the C-terminus produce very short grain (Mao et al., 2010; Takano-Kai et al., 2013). Other grain length QTLs have been identified on chromosome (chr) three using different mapping populations. These include *GW3.1* (Li et al., 2004), *GL3.1/GL3* (Qi et al., 2012), *qGL3* (Zhang et al., 2012), and *TGW3/GL3.3* (Hu et al., 2018; Xia et al., 2018). Several grain length QTLs have also been reported on chr7, including *qGL7* (Bai et al., 2010), *qGL7.2* (Shao et al., 2010), *GLW7/OsSPL13* (Si et al., 2016), *SLG7* (Zhou et al., 2015), and *GL7/GW7* (Wang S. et al., 2015). Copy number variation at the *GL7* locus contributes to grain size diversity in *O. sativa* (Wang Y. et al., 2015).

Numerous Grain width and grain weight loci are also found on different chromosomes, including *GW2* (Song et al., 2007), *GIF1* (Wang et al., 2008), *GW5*/*qGW5/SW5*, (Shomura et al., 2008; Weng et al., 2008), *GS5* (Li et al., 2011), *GW6a* (Song et al., 2015), *TGW6* (Ishimaru et al., 2013), *OsSPL16/GW7* (Wang S. et al., 2015), and *GW8*/*OsSPL16* (Wang et al., 2012).

Haplotype characterization approach is a powerful tool that help in understanding a particular germplasm and identify and validate trait SNPs for applied breeding purposes. High-throughput SNP markers were identified and validated for semi-dwarf gene *sd1* (Angira et al., 2019), Cercospora disease locus *CRSP2.1* (Addison et al., 2021), and an aroma gene *BADH2* (Addison et al., 2020) of rice using haplotype characterization approach in the recent studies. Similar haplotype analysis approaches were reported in other crops (Maldonado et al., 2019; Brinton et al., 2020; Bruce et al., 2020; Marsh et al., 2022) and proven to be vital in understanding the trait genetic architecture in a particular germplasm. We developed KASP (Kompetitive allele specific polymerase) SNP markers using the haplotype characterization approach. KASP is an allele-specific oligo extension and fluorescence-based cost-effective genotyping technology (Kumpatla et al., 2012; Semagn et al., 2014). In this study, we identified and validated SNPs for the identified loci and developed the SNPs as KASP assays to be used in an applied breeding program.

## Materials and Methods

### United States Germplasm Panel

A United States rice germplasm panel (U.S. germplasm panel) was compiled to characterize grain shape phenotypes. Additionally, this panel was used to discover and validate grain shape loci and trait markers. The panel consists of 323 lines and represents the genetic diversity present in modern and elite southern U.S. rice germplasm. The panel consists of key historical varieties and germplasm, advanced breeding lines, and modern released varieties from the rice breeding programs of Louisiana, Arkansas, Texas, Mississippi, California, and Missouri.

### “Trenasse” x ‘Jupiter’ Recombinant Inbred Line Population

A bi-parental recombinant inbred line (RIL) population consisting of 286 RILs was developed from a cross between the varieties Trenasse (T) and Jupiter (J) and is referred to as the ‘TJ RIL’ population. Trenasse is a typical U.S. long-grain variety (Linscombe et al., 2006), and Jupiter is a typical U.S. medium-grain variety (Sha et al., 2006). The TJ RIL population was developed using single seed decent breeding method and derived at the F8 generation. Jupiter is widely grown medium grain variety in the southern United State and Trenasse, released in 2006 and still exists in the pedigree of modern long grain varieties. The reported average yield of Trenasse and Jupiter was 8089 kg ha^−1^ across 21 statewide trials during 2001–2003 (Linscombe et al., 2006) and 8734 kg ha^−1^ across 37 statewide trials during 2002–2004 (Sha et al., 2006), respectively.

### Public Database

For this study, we used the International Rice Informatics Consortium SNP-Seek (IRRI SNP-Seek) database available at snp-seek.irri.org (Alexandrov et al., 2014; Mansueto et al., 2016, 2017). The SNP-Seek portal hosts multiple genomic datasets and formats including the sequencing data of the 3,000 rice genomes project (3K RGP) (Li et al., 2014) and the High-Density Rice Array (HDRA) dataset (McCouch et al., 2016). The HDRA dataset contains 700,000 SNPs (single nucleotide polymorphism) genotyped on 1,568 diverse rice accessions, including 92 U.S. rice varieties (McCouch et al., 2016). Of the five SNP sets available for panel—All (32M), All biallelic (29M), Base (18M), Filtered (4.8M), and Core (404k) (Mansueto et al., 2017)—this study utilized the 18M SNP set (3KBase) as well as the HDRA dataset to characterize haplotypes in the U.S. germplasm diversity panel.

### Phenotyping Methods

The TJ RIL population (*n* = 286) was planted at the H. Rouse Caffey Rice Research Station (HRCRRS) near Crowley, LA in 2017 and 2018 and phenotyped for grain traits–grain length and grain width. The U.S. germplasm panel was planted at the HRCRRS in 2019 and phenotyped for grain traits. The panel was phenotyped only for 1 year because the grain dimension traits are highly heritable and the environment does not have a significant effect on the traits (Fan et al., 2006; Gao et al., 2016). Both the TJ RILs and the U.S. germplasm panels were planted with a Hege seed drill, and each row was 1.8 m in length. Standard agronomic practices were followed according to the Louisiana Rice Production handbook (LSU Agcenter, 2014). The SeedCount SC6000R - Reflectance Image Analysis System (nextinstruments.net) was used to phenotype grain traits: length, width, and length/width ratio (LWR). The instrument uses software and flatbed scanner technology to rapidly and accurately analyze the physical characteristics of a grain sample. The instrument reliably eliminates broken and double grains in a scanning plate.

### Quantitative Trait Loci Mapping for Grain Dimension Traits

Quantitative trait loci mapping was conducted using the TJ RIL population. The population was genotyped using KASP SNP assays at the HRCRRS. Linkage map construction and QTL mapping of grain shape traits were executed using composite interval Mapping implemented in QTL IciMapping software V4.1 (Meng et al., 2015). The Kosambi mapping function was used for linkage map development, and the significant logarithm of the odds (LOD) threshold was calculated using 1000 permutations and Type I error rate. Additive QTLs were detected using a 1.0 cM stepwise regression with a 0.001 probability threshold.

### Haplotype Characterization of Candidate Genes Across the 3K RGP Panel

Single nucleotide polymorphism data for the *GS3* and *GL7* genes were extracted from the IRRI SNP-Seek database using the 3K RGP dataset and 3kbase dataset filters (iric.irri.org; snp-seek.irri.org) (Alexandrov et al., 2014; Mansueto et al., 2017). The dataset consisted of 3,024 genotypes representing the global diversity of rice germplasm including *aus*, *indica*, *indica admix*, *japonica admix, tropical japonica*, *temperate japonica*, and *aromatic* subpopulation groups. However, modern U.S. germplasm is not well represented in this dataset, which contains only 32 U.S. varieties. Therefore, the haplotypes identified in the 3K RGP diversity panel have to be surveyed across the U.S. rice germplasm panel.

SNP data for the *GS3* gene (*LOC_OS03G0407400*), located at 16,729,501–16,735,109 base pair (bp) (IRGSP v.1) on chr3, consisted of 169 SNPs identified in the 3,024 genotypes available in the SNP-Seek portal. Heterozygous SNP calls were considered as missing data. Single nucleotide polymorphisms having missing data >0.2 across the 3,024 germplasm samples were omitted from the analysis. Next, samples having >0.2 missing SNP calls were removed from further analysis, and then SNPs with minor allele frequency <0.001 were omitted. After these filtering steps, 2,426 samples remained, and these had genotyping data at 96 SNPs, which were organized into haplotype groups. A haplotype is defined as having identical alleles at all 96 SNP loci. A haplotype number was assigned to haplotypes found in more than 30 samples (>0.012); haplotypes found in less than 30 samples were considered rare haplotypes. This SNP haplotype characterization identified eight *GS3* haplotypes, which accounted for 92% of the 2,426 samples.

Similar haplotype characterization steps were followed for the *GL7* grain shape gene. Single nucleotide polymorphism data for the gene *GL7* (LOC_Os07g41200), located at 24,664,168–24,669,324 bp (IRGSP v.1) on chr7, consisted of 82 SNPs identified in the 3,024 genotypes available in the SNP-Seek portal. Heterozygous SNP calls were considered as missing data. Single nucleotide polymorphisms having >0.2 missing data across 3,024 germplasm samples were omitted. Next, samples having >0.2 missing data across the remaining SNPs were removed from further consideration. In the final filtering step, SNPs having minor allele frequency <0.001 were omitted. Following these filtering steps, 2,993 samples remained, and they had genotyping data at 71 SNPs which were then assigned to haplotype groups. Haplotypes present in less than 35 (<0.012) samples were classified as rare haplotypes. Samples that could not be classified into haplotype groups due to missing data were assigned to the Unclassified group. Nine haplotype groups accounted for 96% of the 2,993 samples. Similar haplotype characterization methods in the 3K RGP panel were previously reported by Angira et al. (2019) and Addison et al. (2021).

### Haplotype Characterization of Identified Grain Length Genes Across U.S. Germplasm

Upon assigning haplotypes for the *GS3* and *GL7* genes in the 3K RGP panel, a set of minimum SNPs was developed that accurately differentiated all haplotypes. The minimum SNP sets consisted of seven and eight SNPs for *GS3* and *GL7* genes, respectively. Flanking sequences (100 bp each side) for each SNP were extracted using the Ricebase database (Edwards et al., 2016), and these SNPs were developed into KASP assays for screening across the U.S. germplasm panel. Kompetitive allele specific polymerase chain reaction genotyping was conducted on the U.S. germplasm panel at the HRCRRS following protocols developed for the LGC SNPline system (biosearchtech.com/products/instruments-and-consumables/genotyping-instruments/snpline-genotyping-automation, accessed 22 October 2021). The minimum SNP set markers were screened across the U.S. germplasm panel for both genes to document the frequency of the haplotype groups characterized using the 3K RGP panel.

### Haplotype Characterization of the *qGL7.1* QTL Region in HDRA Dataset

To explore haplotype variation across the more extensive *qGL7.1* QTL region, SNP data were extracted from the HDRA dataset using the SNP-Seek portal. The dataset consisted of 1,568 germplasm samples, and 763 SNPs were detected in the 475 Kb *qGL7.1* QTL region. To explore haplotype variation only in modern U.S. germplasm, we focused on the 34 U.S. genotypes in the HDRA dataset that were released after 1980. Single nucleotide polymorphisms having >0.15 missing data across the 34 U.S. samples, samples having >0.2 missing data across the SNPs, SNPs with minor allele frequency <0.02, and monomorphic SNPs were omitted from the further analysis. After these filtering steps, genotyping data for 358 SNPs on 34 U.S. lines remained for analysis. Haplotypes at a frequency of ≥0.05 were characterized in this dataset and a minimum SNP set was developed that could accurately classify all of the samples into the correct haplotype groups. This minimum SNP set was developed into KASP assays and screened across the U.S. germplasm panel.

### Interaction of *GS3* and *qGL7.1* in the TJ RIL Population, Segregating Populations, and Advanced Breeding Lines

The TJ RIL population was used to examine the interaction between the *GS3* and *qGL7.1* genes. The RILs were classified into four classes—Class 1: *GS3*-Long/*qGL7.1*-Long; Class 2: *GS3*-Long/*qGL7.1*-Med; Class 3: *GS3*-Med/*qGL7.1*-Long; and Class 4: *GS3*-Med/*qGL7.1*-Med, based on the alleles at both loci. Grain length phenotypes were correlated with these classes to understand the effect of the combination of alleles at both loci. We also used 78 advanced breeding lines that were developed by crossing long- and medium-grain varieties. These varieties were phenotyped for grain length and grain width, in addition to yield, for 2 years.

## Results

### Quantitative Trait Loci Mapping for Grain Dimension Traits

Quantitative trait loci (QTL) mapping was conducted for grain dimension traits using 286 RILs derived from a cross between Trenasse (long-grain) and Jupiter (medium-grain) based on phenotype data collected over 2 years. The population was genotyped with 139 KASP SNPs, and a genetic linkage map of 1547 cM was constructed. Two major QTLs were detected for grain length (*qGL3.1* and *qGL7.1*), grain width (*qGW3.1* and *qGW7.1*), and LWR (*qGLWR3.1* and *qGLWR7.1*). The long-grain parent, Trenasse, contributed the alleles for increased grain length and LWR at both QTLs. The medium-grain parent, Jupiter, contributed the alleles for increased grain width at both grain width QTLs. All QTLs were detected within and across both years. Major QTLs for grain length, *qGL3.1* and *qGL7.1*, explained about 53% and 22% of phenotypic variation, respectively (Table 1).

**TABLE 1:** Grain dimension Quantitative trait loci (QTL) detected in the Trenasse/Jupiter Recombinant Inbred Line population.

Trait	QTL name	chr	Pos (cM)	QTL interval start			QTL interval stop			LOD	PVE	Add	Donor^a^
				Marker name	Pos (cM)	IRGSP V.1	Marker name	Pos (cM)	IRGSP V.1				
Length	*qGL3.1*	3	89	LSU-963	88.58	16,733,441	LSU-92	111.08	23,999,431	74.91	53.34	−0.36	Trenasse
Length	*qGL5.1*	5	63	LSU-48	46.58	16,473,206	LSU-329	66.57	19,504,928	5.03	2.67	0.08	Jupiter
Length	*qGL7.1*	7	124	LSU-323	124.00	24,845,026	LSU-159	125.84	25,319,809	43.00	22.36	−0.23	Trenasse
Width	*qGW3.1*	3	49	LSU-1253	48.04	9,828,999	LSU-309	53.52	10,500,404	6.21	4.11	0.03	Jupiter
Width	*qGW3.2*	3	89	LSU-963	88.58	16,733,441	LSU-92	111.08	23,999,431	18.60	13.11	0.06	Jupiter
Width	*qGW3.3*	3	177	LSU-10	171.96	32,674,916	LSU-240	178.10	34,454,081	6.10	4.14	0.03	Jupiter
Width	*qGW7.1*	7	124	LSU-323	124.00	24,845,026	LSU-159	125.84	25,319,809	39.92	32.70	0.09	Jupiter
Width	*qGW8.1*	8	200	LSU-445	199.62	18,966,446	LSU-481	214.61	21,589,285	9.29	6.01	−0.04	Trenasse
Width	*qGW12.1*	12	81	LSU-1266	70.13	24,395,807	LSU-265	81.52	26,133,920	5.99	3.80	0.03	Jupiter
LWR	*qLWR3.1*	3	49	LSU-1253	48.04	9,828,999	LSU-309	53.52	10,500,404	5.99	2.28	−0.05	Trenasse
LWR	*qLWR3.2*	3	89	LSU-963	88.58	16,733,441	LSU-92	111.08	23,999,431	63.29	38.24	−0.19	Trenasse
LWR	*qLWR5.1*	5	14	LSU-587	12.11	3,341,107	LSU-340	28.11	6,607,675	6.42	2.63	0.05	Jupiter
LWR	*qLWR7.1*	7	124	LSU-323	124.00	24,845,026	LSU-159	125.84	25,319,809	58.98	33.12	−0.18	Trenasse
LWR	*qLWR8.1*	8	203	LSU-445	199.62	18,966,446	LSU-481	214.61	21,589,285	7.13	3.07	0.06	Jupiter

A total of 286 RILs, were phentotyped for grain length and grain width for 2 years and genotyped with 139 Kompetitive Allele-Specific PCR (KASP) single nucleotide polymorphism (SNP) markers. ICIMapping software was used to map the QTLs.

LOD, logarithm of the odds; PVE, percent variation explained; Add = additive effect; Pos = position; chr = chromosome.

a Donor indicates the parent that contributed the allele for the increased value of the trait in the Jupiter/Trenasse RIL, population.

The QTLs detected for the three traits co-localized to the same two regions on chr3 and chr7, with both regions encompassing previously identified grain shape genes in rice. The grain dimension QTLs detected on chr3 were mapped between 16.73 and 24.00 Mb, a region that harbors the major grain size gene *GS3* (Fan et al., 2006, 2009; Takano-Kai et al., 2009). The QTLs detected on chr7 were mapped between 24.85 and 25.32 Mb, which is 176 kb from the closest flanking marker of the *GL7* locus (Wang Y. et al., 2015). Grain dimension traits (grain length, grain width, and LWR) were highly correlated and the QTLs for both the traits were co-localized. Thus, we focused on only grain length trait in this study. However, we explored all the reported grain shape traits in the QTLs. To investigate whether the genes *GS3* and *GL7* underlie the QTLs detected in this study, *qGL3.1* and *qGL7.1*, respectively, the haplotypes of each gene were characterized using the global rice diversity 3K RGP panel and evaluated across the U.S. germplasm panel that had been phenotyped for grain dimension traits.

### Haplotype Characterization of Candidate Genes Across the 3K RGP Panel

The IRRI SNP-Seek database contains re-sequencing data for 3,024 diverse rice samples (3K RGP panel). However, the U.S. rice germplasm is not well represented in this panel. To leverage the 3K RGP re-sequencing data for use evaluating our specific germplasm of interest, we first characterized the haplotype diversity of the *GS3* and *GL7* genes across the 3K RGP panel and identified the minimum set of SNPs necessary to distinguish the haplotypes of each gene. These minimum sets of SNPs for both genes were subsequently used to develop KASP SNP assays to evaluate the TJ RIL mapping population and the U.S. rice germplasm diversity panel.

### Haplotype Characterization of the GS3 Gene Across the 3K RGP Panel

**FIGURE 1:**
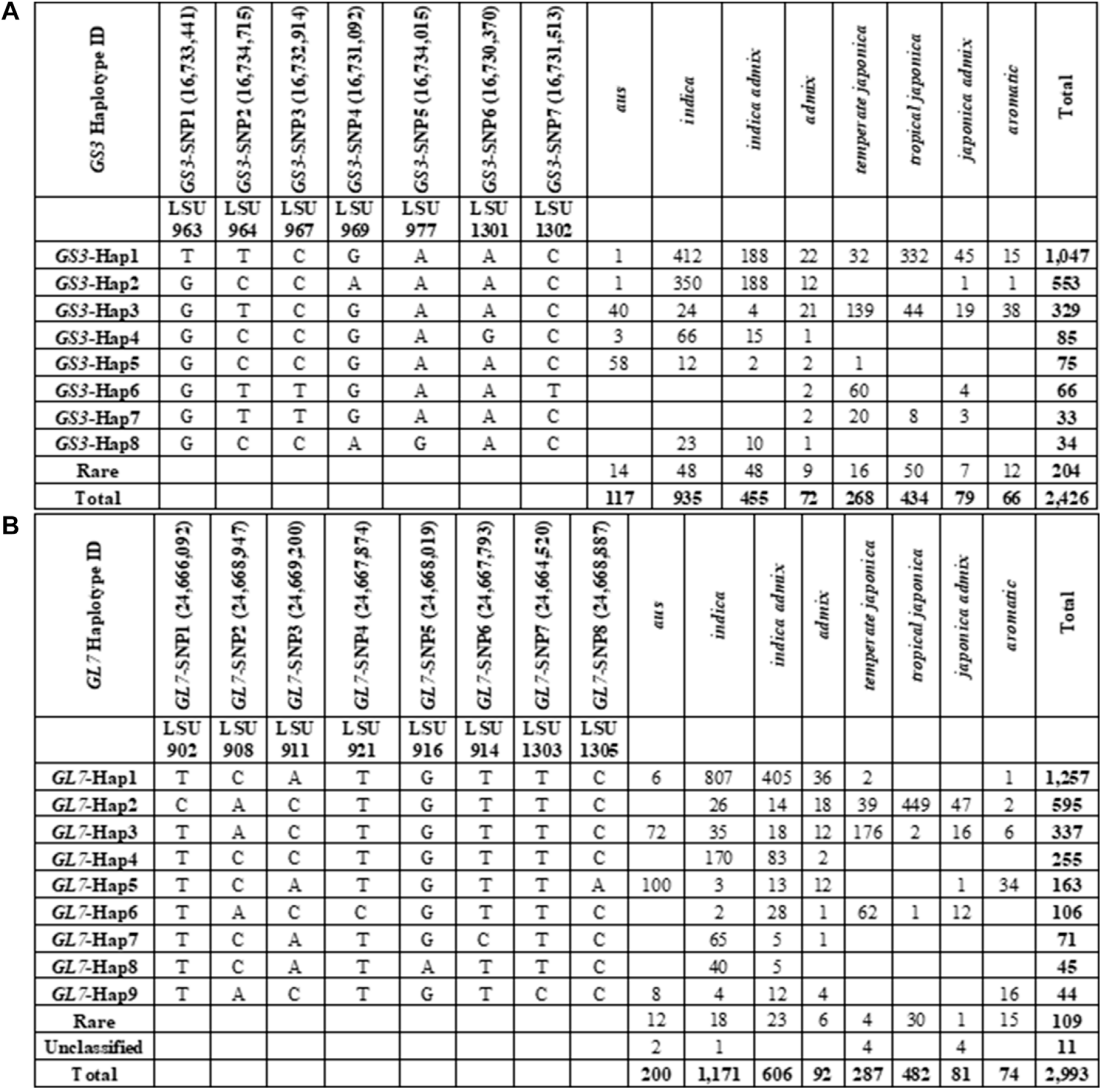
SNP data was obtained from SNP-Seek online portal of the International Rice Research Institute for *GS3* and *GL7* genes and haplotypes were characterized based on specified criteria with each gene. **(A)** Haplotype characterization of the *GS3* gene in IRRI SNP-Seek 3K RGP genotype data. **(B)** Haplotype characterization of the *GL7* gene in IRRI SNP-Seek 3K RGP genotype data.

A total of 169 SNPs were identified in the *GS3* gene (*LOC_OS03G0407400*) based on the re-sequencing data for the 3K RGP panel. After filtering SNPs and genotypes based on missing data (>0.2) and minor allele frequency (<0.001), a dataset was extracted consisting of genotypes for 2,426 samples evaluated at 96 SNP loci (Figure 1A, Supplementary Table S1). Based on these 96 SNPs, eight haplotype groups were identified with a frequency of >0.012. These eight haplotypes described the variation in *GS3* found in 2,222 (92%) of the samples. Haplotypes in 204 samples (8%) could either not be classified due to missing data or were rare; these samples were omitted from further analysis. Based on the haplotype assignments from the 96 SNPs, a subset of seven SNPs (minimum SNP set) was identified that could accurately differentiate the eight haplotype groups and correctly assign samples to each group (Figure 1A). This minimum SNP set was used to represent the haplotype diversity in the *GS3* gene.

For this study, we were most interested in haplotypes that could be used to differentiate *Japonica* samples, as U.S. rice germplasm is predominantly of *Japonica* ancestry. *GS3-*Hap1 was the most common SNP haplotype across the 3K RGP panel, found in 1,047 samples, of which about 58% were of *Indica* ancestry (*n* = 601), 40% were of *Japonica* ancestry (*n* = 424), and 2% were admixtures (*n* = 22) (Figure 1A, Supplementary Table S1). Within the *tropical japonica* subpopulation, *GS3-*Hap1 was the predominant haplotype, accounting for 76% (*n* = 332) of *tropical japonica* genotypes. *GS3-*Hap1 was distinguished from all other haplotypes by the previously reported casual C-A mutation allele in the second exon of *GS3* at 16,733,441 bp (*GS3*-SNP1), containing the long-grain “T” allele (Fan et al., 2009; Takano-Kai et al., 2009). *GS3-*Hap3 was present in 329 genotypes, of which 73% belonged to the *Japonica* varietal group (*n* = 240), 21% were *Indica* (*n* = 68), and 6% were admixed (*n* = 21). *GS3-*Hap3 was the third most common haplotype across the 3K RGP panel and the predominant haplotype within the *temperate japonica* subpopulation, accounting for 52% (*n* = 139) of all *temperate japonica* lines (Figure 1A, Supplementary Table S1). Based on the association of *GS3* haplotypes to specific subpopulations, we anticipated that the *GS3*-Hap1 allele would be predominant in U.S. long-grain germplasm and that *GS3*-Hap3 would be predominant in U.S. medium-grain germplasm. Other common haplotypes observed in the 3K RGP panel were predominant in samples of *Indica* ancestry and were rare or not present in *Japonica* samples.

### Haplotype Characterization of the GL7 Gene Across 3K RGP Panel

A total of 78 SNPs were identified in the *GL7* gene (LOC_Os07g41200) based on the re-sequencing data for the 3K RGP panel. After filtering SNPs and genotypes based on missing data (>0.2) and minor allele frequency (<0.001), a dataset consisting of genotypes for 2,993 samples evaluated at 71 SNPs was extracted. Based on these 71 SNPs, nine haplotypes were identified with a frequency >0.012. These nine haplotypes described the variation in *GL7* found in 2,873 (96%) samples (Figure 1B, Supplementary Table S2). Haplotypes in 120 samples (4%) could either not be classified due to missing data or were rare (<1.5%), so these samples were omitted from further analysis. Based on the haplotype assignments from the 71 SNPs, a subset of eight SNPs (minimum SNP set) was identified that could accurately differentiate the nine haplotype groups. This minimum SNP set was used to represent haplotype variation in the *GL7* gene (Figure 1B).

*GL7*-Hap1 was the most common SNP haplotype in the 3K RGP panel and was found in 1,257 samples, of which 97% were of *Indica* ancestry and about 3% were admixtures. *GL7*-Hap2 was present in 595 samples, of which 90% belonged to the *Japonica* varietal group, 7% were *Indica*, and the remaining 3% were admixed (Figure 1B, Supplementary Table S2). *GL7*-Hap2 was the second most common haplotype group in the panel and the most common haplotype in *Japonica* samples. *GL7-*Hap2 was present in 93% of *tropical japonica* samples in the 3K RGP panel. *GL7-*Hap3 was present in 337 samples, of which 52% belonged to the *temperate japonica* subpopulation. *GL7*-Hap3 was the predominant haplotype within *temperate japonica*, accounting for 61% of all *temperate japonica* samples. Based on these observations, we anticipated that the *GL7-*Hap2 would be the most common haplotype within U.S. long-grain germplasm and *GL7*-Hap3 would predominate within U.S. medium-grain germplasm.

### Haplotype Characterization of *GS3* and *GL7* in U.S. Germplasm

The haplotypes of *GS3* and *GL7* observed in the 3K RGP panel were investigated in the U.S. germplasm panel to determine whether they underlie the *qGS3.1* and *qGL7.1* QTLs detected in the TJ RIL population. The minimum SNP sets that differentiated *GS3* (7 SNPs) and *GL7* (8 SNPs) haplotypes were converted into KASP assays and used to screen the U.S. germplasm panel.

**FIGURE 2:**
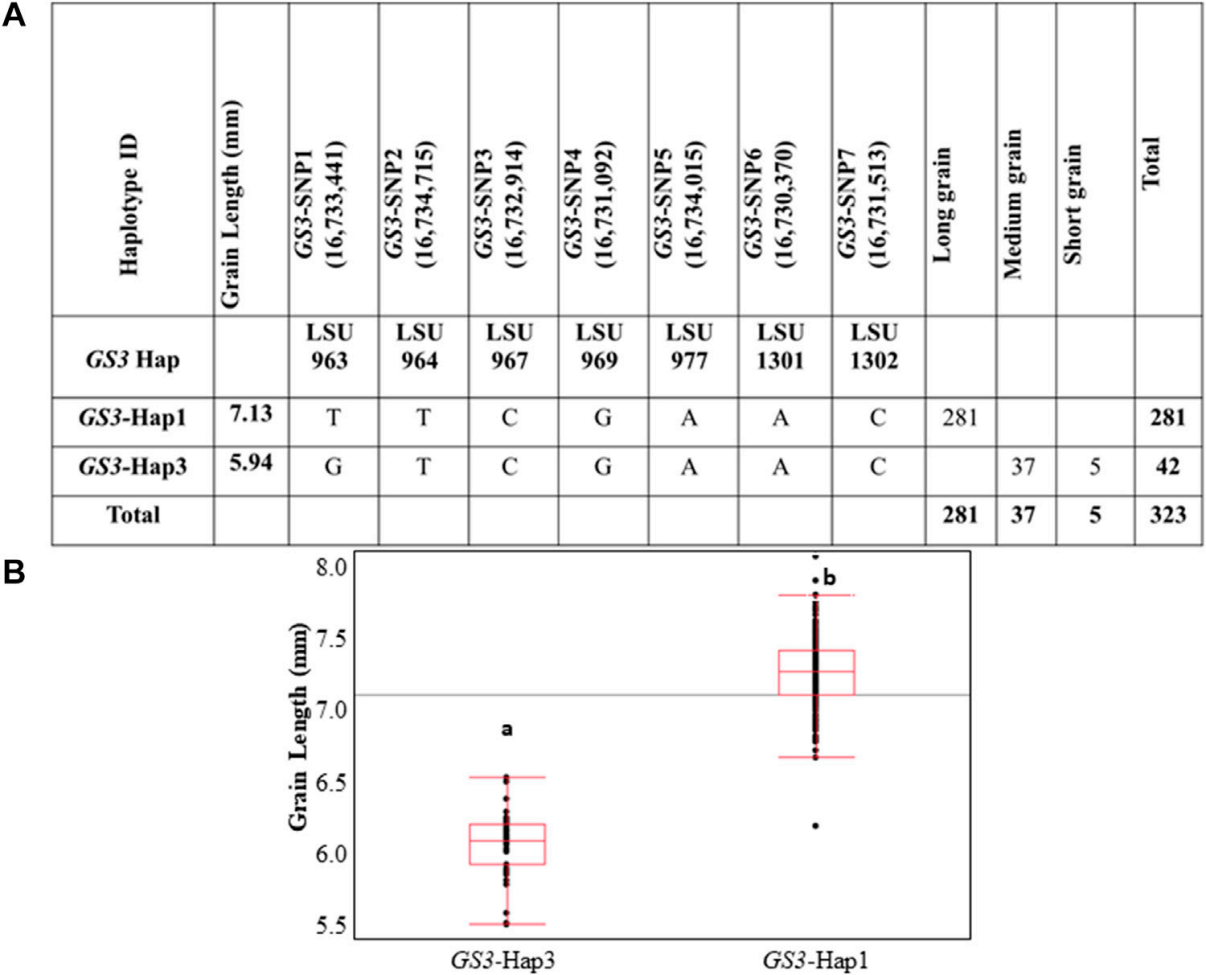
**(A)** Haplotype characterization of *GS3* in U.S. rice germplasm. Haplotypes that were characterized in the IRRI 3K RGP panel were screened in U.S. rice germplasm panel based on the minimum SNP set identified to differentiate the eight haplotype groups in IRRI 3K RGP dataset. **(B)** Phenotypic association of haplotype/SNP alleles and grain length across U.S. rice germplasm. Different alphabet letters on each class represent the statistically significant difference *p* < 0.001 within each graph.

Of the eight *GS3* haplotypes observed in the 3K RGP panel, only two haplotypes were observed within the U.S. rice germplasm panel (Figure 2A, Supplementary Table S3). *GS3*-Hap1 was present in 281 genotypes, all of which were previously classified as long-grain germplasm. *GS3*-Hap3 was present in 42 genotypes, all were previously classified as medium or short grain germplasm. A comparison of grain length in samples carrying *GS3*-Hap1 or *GS3*-Hap3 was highly significant (*p* < 0.0001). The average grain length of the entire panel was 6.98 mm, with a standard deviation of 0.47 mm, and a range of 5.37–7.95 mm. The *GS3*-Hap1 group had an average grain length of 7.13 mm, while the *GS3*-Hap3 group had an average length of 5.94 mm. *GS3*-Hap1 and *GS3*-Hap3 explained ∼75% of grain length variation in the U.S. germplasm panel and accurately differentiated long- and medium-grain varieties (Figures 2A,B). Thus, we concluded that the *GS3* gene underlies the *qGS3.1* QTL and that it is a major gene underlying grain length variation in U.S. germplasm, accurately distinguishing grain market classes. The SNP *GS3*-SNP1, which represents the known casual C-A point mutation in the *GS3* gene, accurately differentiates long- and medium-grain varieties and is an ideal functional SNP for use as a trait marker.

Of the nine *GL7* haplotypes observed in the 3K RGP panel, only two haplotypes (*GL7*-Hap2 and *GL7*-Hap3) were present in U.S. rice germplasm (Supplementary Table S3). *GL7*-Hap2 was present in 98% of the U.S. panel, including 100% of long-grain varieties and 86% of medium/short-grain varieties. Only six U.S. varieties (S-201, S-102, M-206, Acadia, Pirogue, and Vista) contained the *GL7*-Hap3 allele, all of which were either California medium-grain varieties and/or historical varieties. No modern Southern U.S. medium-grain variety contained *GL7*-Hap3. The parents of the TJ RIL population contained *GL7*-Hap2 at the *GL7* gene, as did all the individual RILs, demonstrating that *GL7* is not the gene underlying the QTL *qGL7.1* detected in this study.

### Haplotype Characterization of the *qGL7.1* QTL Region Using the HDRA Dataset

Upon concluding that the candidate gene *GL7* did not underlie the QTL *qGL7.1*, haplotype analysis was conducted across the *qGL7.1* QTL region. The QTL was mapped to a region of 474,783 bp (1.84 cM) on chr7, which was too large to conduct haplotype analysis based on re-sequencing data. We therefore utilized the HDRA dataset (McCouch et al., 2016) and identified 763 SNPs across the QTL region based on genotypes from 1,568 samples. To prioritize the identification and characterization of the haplotypes most likely to be present in modern U.S. rice germplasm, we focused on the 34 U.S. varieties in the HDRA dataset that were released after 1980. Single nucleotide polymorphisms having >0.15 missing data across 34 U.S. varieties, samples with >0.2 missing data across the SNPs, SNPs with minor allele frequency <0.02, and monomorphic SNPs were excluded from the analysis. Upon filtering of SNPs, 358 SNPs remained for haplotype characterization across 34 U.S. lines. Based on these 358 SNPs, four haplotype groups were identified at a frequency of ≥0.05. A minimum set of three SNPs was identified that accurately differentiated the four haplotypes. The four haplotypes were perfectly associated with the previously defined grain classes. *qGL7.1*-Hap1 (*n* = 13; 38%) and *qGL7.1*-Hap2 (*n* = 2; 6%) were present in samples classified as long grain, where *qGL7.1*-Hap3 (*n* = 7; 21%) and *qGL7.1*-Hap4 (*n* = 2; 0.1%) were present in samples classified as medium/short grain (Supplementary Figure S1A; Supplementary Table S4).

The three SNPs that differentiated the *qGL7.1* haplotypes were developed into KASP assays and surveyed across the entire U.S. germplasm panel. Three of the four haplotypes (*qGL7.*1-Hap1, *qGL7.*1-Hap3, and *qGL7.*1-Hap4) were detected in modern U.S. germplasm. The most frequent haplotype was *qGL7.*1-Hap1 (*n* = 271; 83.9%), found in 266 long-grain and five medium-grain varieties. Haplotype *qGL7.*1-Hap3 (*n* = 43; 13.3%) was observed in 14 long-grain, 27 medium-grain, and two short-grain samples, and was the predominant haplotype in medium grains. *qGL7.*1-Hap4 (n = 5; 1.5%) was observed in two long-grain, one medium-grain, one short-grain samples, and was rare in southern U.S. rice germplasm. Four samples (1.2%) had unclassified haplotypes due to heterozygous SNP calls (Supplementary Figure S1B; Supplementary Table S5). Together, *qGL7.1* haplotypes explained 46% of the variation for grain length across the U.S. germplasm panel. *qGL7.*1-Hap1 was associated with long-grain length (7.1 mm) that was significantly longer than either *qGL7.*1-Hap3 (6.3 mm) or *qGL7.*1-Hap4 (5.6 mm).

**FIGURE 3:**
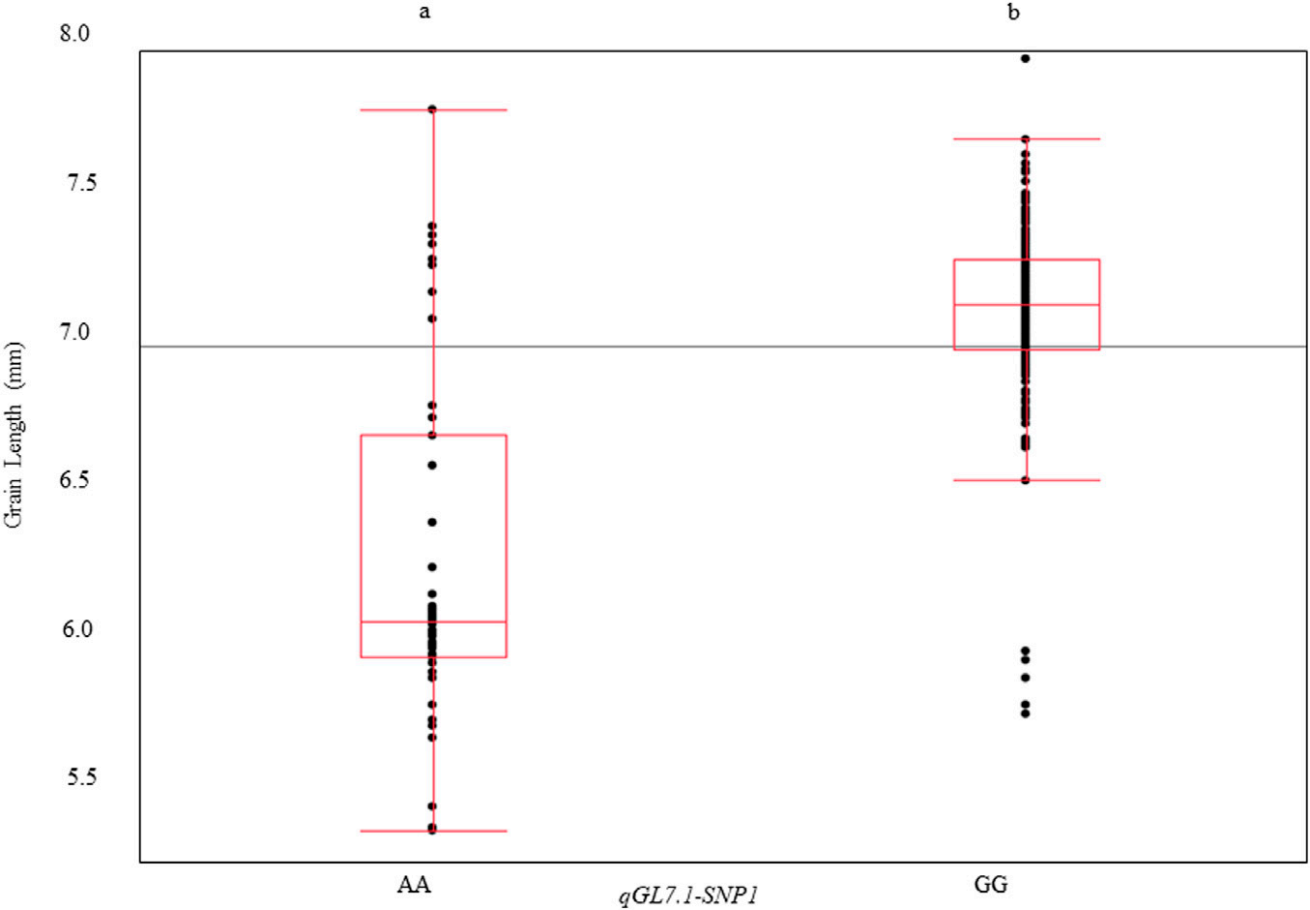
Grain length variation explained by *qGL7.1*-SNP1 (LSU-1480) across a U.S. rice germplasm panel. *qGL7.1-SNP1* was identified in the HDRA dataset that was obtained from SNP-Seek online portal within the *qGL7.1* QTL region. QTL *qGL7.1* was detected on Chromosome seven in Trenasse/Jupiter RIL population. *qGL7.1-SNP1* differentiated long-grain haplotypes from medium-grain haplotypes within U.S. lines of HDRA dataset. Allele A is associated with the medium-grain genotypes and allele G is associated with long-grain genotypes. Different alphabet letters on each class represent the statistically significant difference *p* < 0.001 within each graph.

To identify a single SNP that could reliably distinguish long- and medium-grain varieties for routine use in southern U.S. breeding programs, the three haplotype SNPs were compared. The SNP *qGL7.1-*SNP1 (25,211,630 bp) was able to differentiate *qGL7.*1-Hap1 (predominantly found in long-grain varieties) from *qGL7.*1-Hap3 (predominantly found in medium-grain varieties), but it does not differentiate *qGL7.*1-Hap3 from *qGL7.*1-Hap4. If used together, *GL7.1*-SNP1 and *qGL7.1-*SNP3 can differentiate all four haplotypes found in the U.S. germplasm, but a single SNP capable of differentiating long-grain q*GL7.1*-Hap1 from medium-grain q*GL7.1*-Hap3 would be sufficient for applied breeding programs focusing on the southern United States. When used alone, SNP *qGL7.1-*SNP1 explained 43% (Figure 3) of the phenotypic variation for grain length. We recommend that it be used as a diagnostic marker to identify the long-grain allele at *qGL7.1* in applied breeding applications in the United States.

### Interaction of *GS3* and *qGL7.1* Within a RIL Population, Segregating Populations, and Advanced Breeding Lines

**FIGURE 4:**
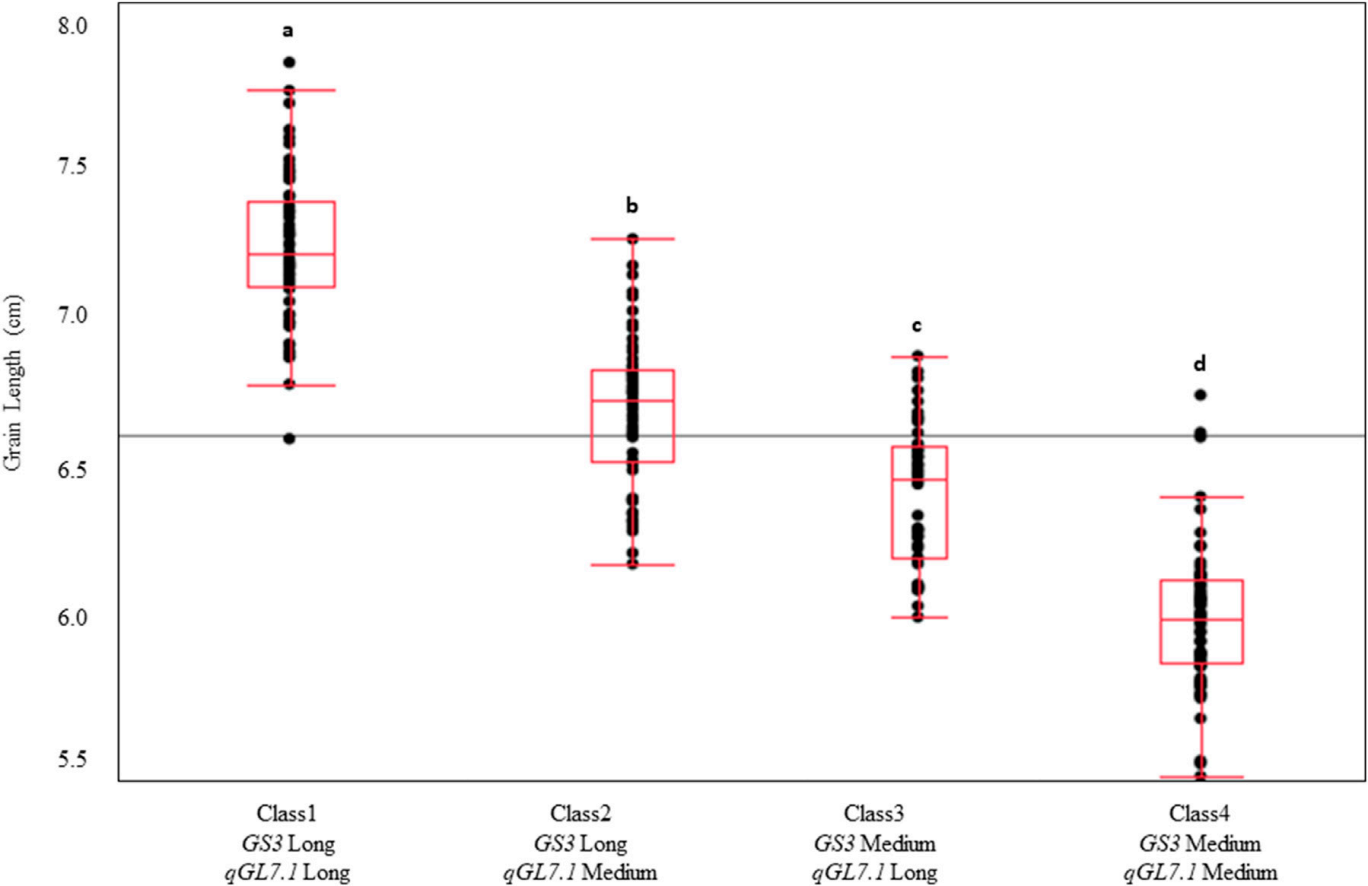
Genotypic classes of Trenasse/Jupiter RILs based on the alleles at *GS3* and *qGL7.1* in the RIL population. Four genotypic classes were identified based on the allele state at *GS3* and *qGL7.1.* Classes denoted by a different letter indicate significant differences between classes (*p* < 0.001).

The allelic states of *GS3* and *qGL7.1* were highly correlated across the U.S. germplasm panel, with 96% of long-grain varieties containing the long-grain allele at both loci and 95% of medium-grain varieties containing the medium-grain allele at both loci. To understand the interaction and effect of each locus in a controlled genetic background, the TJ RILs were grouped into four classes based on alleles at *GS3-SNP1* and *qGL7.1-SNP1*, and these classes were compared for grain length. The classes were defined as Class1: *GS3*-Long/*qGL7.1*-Long; Class2: *GS3*-Long/*qGL7.1*-Med; Class3: *GS3*-Med/*qGL7.1*-Long; Class4: *GS3*-Med/*qGL7.1*-Med. Significant differences in grain length were observed among classes (*p* < 0.001). Class1 (7.2 mm) had the longest grain length and Class4 had the shortest (6.0 mm). Class2 exhibited a longer grain length than Class3, demonstrating that *GS3* has a larger effect than *qGL7.1* in this population (Figure 4). Selecting long-grain alleles at both loci would help develop a longer grain rice variety using U.S. germplasm.

To further validate the effect of *GS3* and *qGL7.1* in different genetic backgrounds, four F_3_ populations (CL272/Mermentau, CL272/Presidio, CRPS/Titan, and Mermentau/Titan) that segregated for both *GS3* and *qGL7.1* loci were examined. The populations were divided into nine genotypic classes based on the allele configurations [homozygous (homo) long (L) allele, heterozygous (het), or homozygous medium (M) allele] at both loci. The genotypic classes were defined as *GS3-*homo L/*qGL7.1*-homo L; *GS3*-homo L/*qGL7.1*-homo M; *GS3*-homo L/*qGL7.1*-het; *GS3*-homo M/*qGL7.1*-homo L; *GS3*-homo M/*qGL7.1*-homo M; *GS3*-homo M/*qGL7.1*-het; *GS3*- het/*qGL7.1*-homo L; *GS3*-het/*qGL7.1*-homo M; and *GS3*-het/*qGL7.1*-het). The average grain length of the genotypic class that was homozygous long at both loci was 7.2 mm, compared to 6.4 mm for the genotypic class that was homozygous medium at both loci. The genotypic class that was heterozygous at both loci was 6.6 mm. The genotypic class containing the homozygous long-grain allele at *GS3* and homozygous medium-grain allele at *qGL7.1* had a longer grain length (7.1 mm) compared to the class containing the homozygous medium-grain allele at *GS3* and homozygous long-grain (6.7 mm) allele at *qGL7.1* (Supplementary Figure S2). Thus, *GS3* again appears to have a larger effect than *qGL7.1* in these additional populations, consistent with observations in the TJ RIL population. Among the lines homozygous for the long-grain *GS3* allele, the allelic state (homozygous long, homozygous medium, heterozygous) at *qGL7.1* did not significantly contribute to grain length. However, among the lines that were homozygous for the medium-grain allele at the *GS3* gene, significantly different grain lengths were observed for the *qGL7.1* allele classes (Supplementary Figure S2).

In addition to the RIL population and segregating populations, we also investigated the effect of these loci on grain length in advanced breeding lines that were developed by crossing long- and medium-grain varieties. Seventy eight breeding lines were genotyped at both loci and phenotyped for grain length across 2 years. These lines were classified into four classes based on allele state at *GS3* and *qGL7.1*. Lines that had the long-grain allele at both loci had an average grain length of 6.9 mm, compared to a grain length of 6.1 mm for lines with the medium-grain allele at both loci. Lines with a long-grain allele at *GS3* and medium-grain allele at *qGL7.1* had an average grain length of 6.5 mm, compared to 6.3 mm for lines that had the medium-grain allele at *GS3* and the long-grain allele at *qGL7.1*. These results confirm that both loci have a significant effect across multiple populations and genetic backgrounds and that *GS3* has a larger effect on grain length than *qGL7.1.*

### Validation and Technical Performance of the Identified Grain Length Trait SNPs

Haplotype characterization and phenotypic association identified a single candidate SNP/locus for use as a trait marker for the major grain size QTLs, *qGS3.1* and *qGL7.1*. For a trait marker to have broad utility in an applied breeding program, it should be informative across the target breeding germplasm and provide high-quality, cost-effective, and reproducible genotype data. *GS3-*SNP1 and *qGL7.1*-SNP1 were validated across the U.S. germplasm panel and shown to be effective at differentiating long- and medium-grain cultivars based on alleles at *GS3* and *qGL7.1*. Thus, these SNPs have potential utility for use in U.S. rice breeding programs. However, for a SNP marker to be effectively deployed in an applied breeding program, it is also essential that the marker provide reproducible results and that it is amenable to high-throughput screening and scoring. To ensure that each of the selected SNPs provides reliable genotyping results for high-throughput molecular breeding activities, the technical performance of the KASP assay was tested. Each assay was tested with three replicates across the U.S. germplasm panel, and a plate of 96 samples from the F_1_ population that included all three genotypic classes was used to evaluate SNP clustering in the presence of heterozygous plants. Each assay provided >99% data return, consistent allele calls across the replicates, and exhibited clear and distinct clusters that could be accurately assessed using auto-scoring (Supplementary Figure S3).

### Investigation Into the Gene Action and Inheritance of *GS3 and qGL7.1*

The four F_3_ populations (CL272/Mermentau, CL272/Presidio, CRPS/Titan, and Mermentau/Titan) segregating for both *GS3* and *qGL7.1* loci were used to investigate the gene action and inheritance of the two loci. Grain length of the lines that were heterozygous at *GS3* and homozygous for the long allele at *qGL7.1* had grain lengths similar to the lines that were homozygous for the medium-grain allele at *GS3* and for the long-grain allele at *qGL7.1*. Similarly, lines that were heterozygous at *GS3* and homozygous for the medium-grain allele at *qGL7.1* had grain lengths similar to lines that were homozygous for the medium alleles at *GS3* and *qGL7.1*. However, lines that were homozygous for the long alleles at *GS3* and *qGL7.1* were significantly longer than lines that were heterozygous at the *GS3* gene and homozygous for the long allele at *qGL7.1* (Supplementary Figure S2). These results demonstrate that the long-grain allele of *GS3* is recessive, consistent with previous reports.

Compared to *GS3*, the gene action of the *qGL7.1* locus was less clear. Among the F_2_:F_3_ lines that were homozygous for the long-grain *GS3* allele, no significant differences in grain length were observed among the three *qGL7.1* genetic classes (homozygous long, heterozygous, and homozygous medium). However, among F_2_:F_3_ lines homozygous for the medium-grain alleles at *GS3*, the heterozygous class at *qGL7.1* was not significantly different from the class that had long-grain allele but was significantly longer from the class that had medium-grain allele at *qGL7.1* (Supplementary Figure S2). These results do not provide sufficient evidence to conclude dominance/recessive nature of *qGL7.1*. Further studies using true F_1_ plants are required to conclude the gene action of *qGL7.1.*

## Discussion

Many genes conferring grain shape have been reported in previous studies. Here, a novel QTL, *qGL7.1*, for grain shape was mapped to a 475 kb region and explained 22% of the phenotypic variation in the TJ RIL population. We explored candidate genes in this region using data available on SNP-Seek platform (Mansueto et al., 2016, 2017). This region harbors 80 genes including 25 expressed proteins, 13 retrotransposons, seven DC1 domain-containing proteins, three C1-like domain-containing proteins, three transposon proteins, two sulfotransferase domain-containing proteins, and two stress responsive A/B barrel domain-containing proteins (Supplementary Table S6). The most promising candidate gene is LOC_Os07g41590, located between 24,931,739–24,933,197 bp. It is a gibberellin (GA) receptor gene, and GAs are reported to be crucial in many developmental processes, including stem elongation, leaf expansion, trichome development, seed germination, pollen maturation, and flowering initiation (Achard and Genschik, 2009). Vaughan et al. (2011) documented the role of GA in determining wheat grain size. They reported that an increase in GA production resulted in up to a 15% increase in grain weight. Therefore, LOC_Os07g41590 may be a grain shape candidate gene underlying the *qGL7.1* QTL. We also explored mutants in the *qGL7.1* region and identified the Giant Embryo (*GE*) gene (Koh et al., 1996; Nagasawa et al., 2013) located at 25.373,984–25,375,652 bp and the *BUI1* (*BENT UPPERMOST INTERNODE1*) gene (Yang et al., 2011) at 25,373,984–25,375,652 bp. *GE* controls cell size in the embryo and cell death in the endosperm. Endogenous *GE* expression is upregulated in mutants, and *GE* overexpression causes a small embryo and enlarged endosperm. Thus, *GE* might be another candidate gene underlying grain length in the *qGL7.1* region. Further investigation is needed to identify other candidate genes in this region that may play a role in grain shape.

The effects of *GS3* and *qGL7.1* on grain length were studied in a wide range of materials including a U.S. germplasm panel (*n* = 323), a RIL (TJ) population (*n* = 286), four F_2_:F_3_ breeding populations, and 78 advanced breeding lines, developed by crossing long and medium-grain parents segregating at both *GS3* and *qGL7.1* loci. In all these materials, both loci have a significant effect on grain length, with *GS3* having a larger effect than *qGL7.1*. Lines carrying the long-grain allele at both loci had the longest grains, and lines carrying medium-grain alleles at both loci exhibited the shortest grains, with other combinations of alleles conferring intermediate grain lengths. These results across a wide range of plant material clearly showed the benefit of using allele-specific markers at both loci to select for grain length in U.S. rice germplasm.

United States long-grain and medium-grain rice varieties differ in their cooking qualities in addition to their grain length. Long-grain rice has an intermediate amylose content of 21–24% and an intermediate gel temperature of 71–74°C as indicated by intermediate alkali spreading value of three–5 (Webb, 1985; Gravois and Webb, 1997; Linscombe, 2017). In contrast, short-to medium-grain rice has a lower amylose content of 15–19% and a low gel temperature of 65–68°C as indicated by alkali spreading value of 6–7 (Webb, 1985; Gravois and Webb, 1997; Linscombe, 2017). Due to these differences in both cooking quality and grain length, it is challenging to cross long- and medium-grain varieties and to select a long- or medium-grain quality line from the segregating population. In this study, we reported high-throughput markers for a major grain length locus *GS3* and a novel QTL *qGL7.1*. Within each grain class (long or medium), these loci do not segregate, and the reported markers may not play any role. However, when crosses are made between long- and medium-grain parents, validated markers *GS3*-SNP1 and *qGL7.1*-SNP1 can play an important role in selecting either long-grain or medium-grain lines. Major waxy alleles are characterized and reported in previous studies (Yang et al., 2019; Shao et al., 2020). Markers are also available for amylose and gel temperature traits. These cooking quality SNP markers along with the validated *GS3*-SNP1 and *qGL7.1*-SNP1 markers for grain length will facilitate breeders’ efforts to select the desired grain class and cooking quality when crossing long- and medium-grain varieties.

The results of this study confirmed that *GS3* is a major grain length gene, and *qGL7.1* is a novel locus on chr7 that significantly affects grain shape in the U.S. germplasm. We identified and validated *GS3*-SNP1 and *qGL7.1*-SNP1 markers which accurately identify long- and medium-grain alleles at *GS3* and *qGL7.1*. Alleles at *GS3* and *qGL7.1* do not segregate within the long- or the medium-grain class, but they do segregate when long- and medium-grain varieties are crossed. Selecting for long-grain alleles at both loci will help increase grain length, and selecting for medium-grain alleles at both loci will facilitate the development of shorter grain varieties. Further studies are needed to investigate the gene action of *qGL7.1* and to discover the genes in the locus.

## Data Availability

The original contributions presented in the study are included in the article/Supplementary Material, further inquiries can be directed to the corresponding authors.

## Supplementary Material

Supplementary Figure 1
**(A)** Haplotype characterization within QTL qGL7.1 region using HDRA SNP-Seek genotype dataset to study the diversity in the QTL region. The haplotype characterization was conducted using only US varieties that were release after 1980. **(B)** Haplotype characterization of the QTL qGL7.1 across US germplasm using minimum SNP set developed from HDRA data within the QTL region.

Supplementary Figure 2 Four segregating F3 populations - CL272/Mermentau, CL272/Presidio, CRPS/Titan, and Mermentau/Titan, that segregated for both GS3 and qGL7.1 genes were used to study the effect of the genes on grain length. Each population had about 70 panicles. A total of 276 panicles were genotyped for GS3 and qGL7.1, and all the seeds of each panicle were phenotyped for grain length. First letter in class name is allele at GS3 gene, and the second letter followed by forward slash is allele state at qGL7.1 gene. L stands for long-grain allele, M stands for medium-grain allele, and H stands for heterozygote state at the gene. **(A)** Average grain length of the defined classes. Different alphabet letters on each class represent the statistically significant difference *p* < 0.001 within each graph.

Supplementary Figure 3 Technical validation of qGS3-SNP1 (left) and qGL7.1 SNP1 (right) in breeding populations segregating at GS3 and qGL7.1. Both clusters for each SNP are distinct and suitable for automated computer scoring.
