# Supplementary figures and images for: Discovery and Validation of Grain Shape Loci in U.S. Rice Germplasm Through Haplotype Characterization
Source: Front Genet. 2022 Sep 12;13:923078. doi: 10.3389/fgene.2022.923078 (PMC9511171; doi:10.3389/fgene.2022.923078)

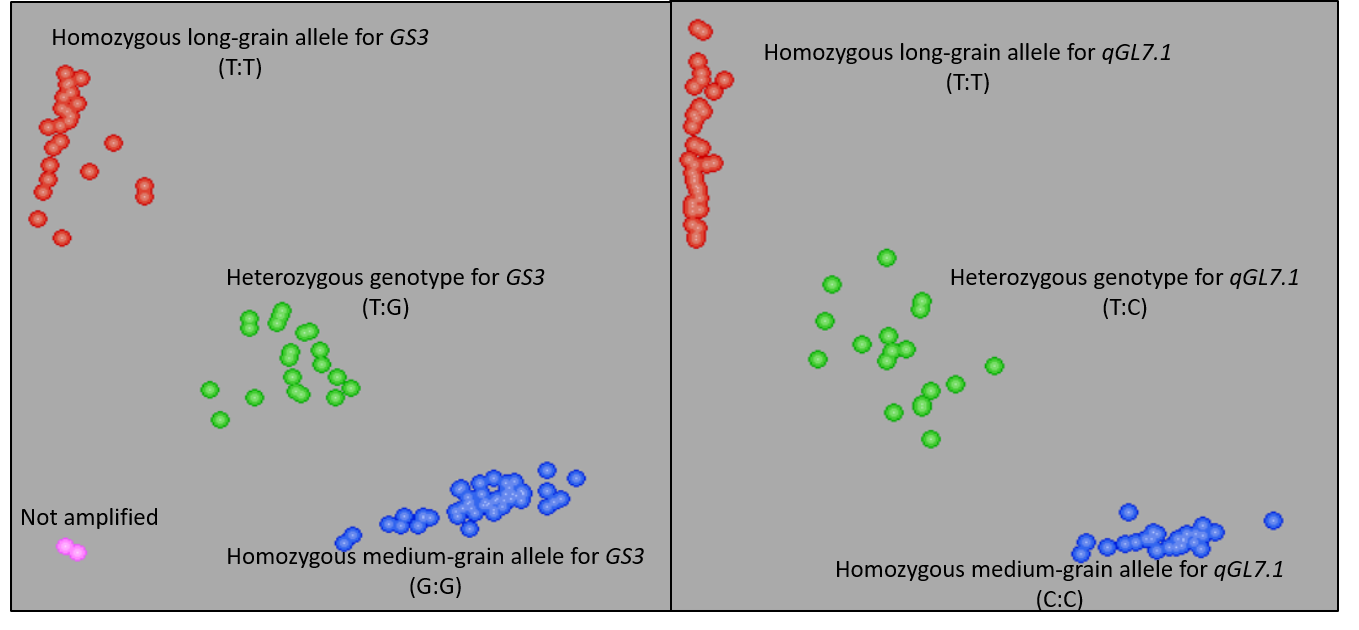

Supplement: Supplementary file 3 [file Image3.TIF]

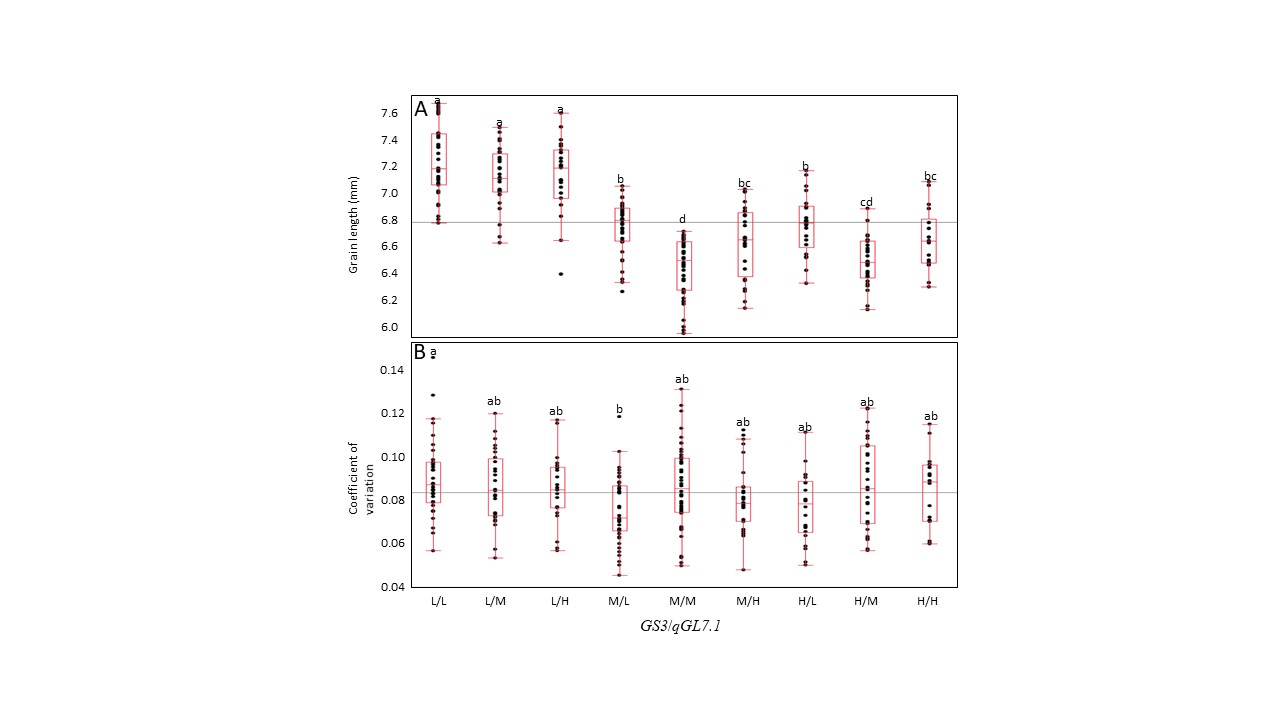

Supplement: Supplementary file 4 [file Image2.tif]

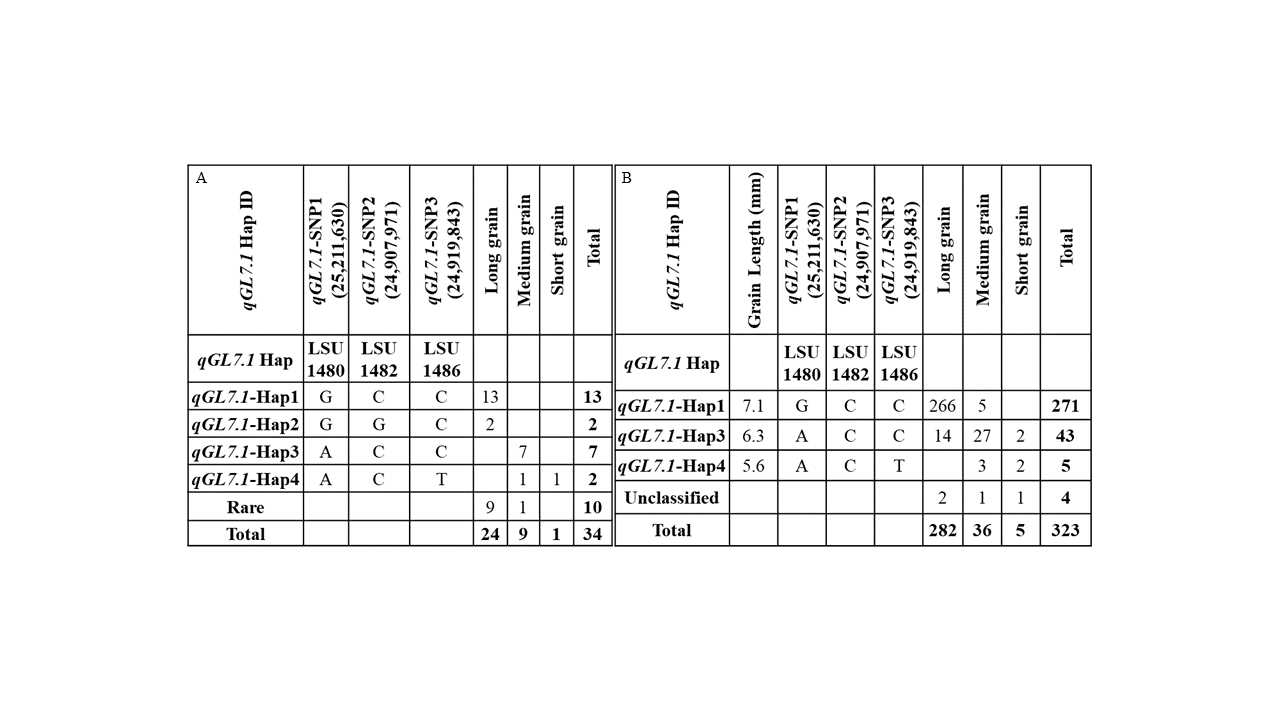

Supplement: Supplementary file 5 [file Image1.tif]
